# Effect of di-(2-ethylhexyl) phthalate (DEHP) on allergic rhinitis

**DOI:** 10.1038/s41598-020-71517-6

**Published:** 2020-09-03

**Authors:** Qi-Yuan Zou, Su-Ling Hong, Hou-Yong Kang, Xia Ke, Xiao-Qiang Wang, Jia Li, Yang Shen

**Affiliations:** Department of Otorhinolaryngology, The First Affiliated Hospital of Chongqing Medical University, 1#Yixueyuan Road, Chongqing, 400016 People’s Republic of China

**Keywords:** Risk factors, Allergy

## Abstract

Allergic rhinitis (AR) is a common chronic inflammatory disease of the upper respiratory tract. Di(2-ethylhexyl) phthalate (DEHP) is a widely used plasticizer and belongs to environmental endocrine disruptors (EDCs). It can be entered the human body which is harmful to health. The relationship between DEHP and AR is still inconclusive. This study aims to investigate the effect of environmental pollutants DEHP on AR. By examining DEHP metabolites in the urine of AR patients and building an AR model. 24 BALB/c mice were used as the study subjects, and ovalbumin (OVA) and DEHP (3 mg/kg/body) were used for intragastric administration. They were divided into control group, DEHP group, OVA group and OVA + DEHP group. Examination, behavioral scoring, inflammatory factor testing, oxidative stress testing, detection of aryl hydrocarbon receptor (AhR) and signaling pathways CYP1A1 and CYP1B1 related proteins and mRNA. The concentrations of 3 metabolites of DEHP (MEHHP, MEOHP, and MEHP) in urine of AR patients were higher. And HE-staining showed that for the control group, many chronic inflammatory cell infiltration and nasal mucosal destruction were observed in the OVA + DEHP group and were more severe than the OVA group. Allergic symptom scores were obtained from sneezing, scratching, number of scratching, and nose flow. The scores of the OVA group and the OVA + DEHP group were higher than 7 points. Serum ELISA and nasal mucosal oxidative stress tests are more serious in the OVA + DEHP group. The expression of AhR protein and its mRNA was increased in the DEHP group, OVA group and OVA + DEHP group. The OVA + DEHP group was more significant in the OVA group and DEHP group. And the mRNAs of the AhR-related signaling pathways CYP1A1 and CYP1B1 were also more prominent in the OVA + DEHP group. DEHP may aggravate its inflammatory response through the AhR pathway closely related to the environment. When combined with OVA, DEHP can further aggravate the OVA-induced nasal inflammatory response and make the nasal cavity have undergone severe changes, and many inflammatory cells have infiltrated. DEHP has shown an adjuvant effect, and the AhR-related signaling pathways CYP1A1 and CYP1B1 may be critical.

## Introduction

Allergic rhinitis (AR) is a common upper respiratory disease. The main clinical symptoms are: paroxysmal sneezing, runny, nasal congestion and nasal itching, which may be accompanied by eye symptoms such as itching and tearing. It is plaguing 10% to 40% of the world's population, endangering human health and reducing the quality of life^1^. The pathogenesis of AR is complex, and that environmental and genetic factors play a decisive role. Environmental factors have become increasingly important in recent years. Not only the atmosphere and water pollution, the study found that environmental endocrine disruptors (EDCs) are closely related to a variety of allergic diseases as a source of environmental pollution of widespread concern worldwide^2^. Di(2-Ethylhexyl) Phthalate (DEHP) is a kind of EDCs, which is the most widely used and widely used plasticizer in China, and that can enter the human body through drinking, eating, skin contact and breathing, and has toxic effects on reproductive development, kidney, respiratory system, immune system and nervous system^3–5^. DEHP and its metabolites have been widely reported which are detected in urine, serum, breast milk and semen, and the detection of DEHP in urine is the most commonly used method^6^. In urine, DEHP will undergo two transformations before excretion, which the mtetabolites mainly include mono-2-ethyl-5-hydroxyhexyl phthalate (MEHHP), mono-2-ethyl-5-carboxypentyl phthalate (MECPP), mono-2-ethyl-5-oxohexyl phthalate (MEOHP), mono-2-ethylhexyl phthalate (MEHP), and mono[2-(carboxymethyl)hexyl] Phthalate (MCMHP), so by measuring the concentration of MEHHP, MECPP, MEOHP, MEHP, MCMHP, the metabolism of DEHP can be understood in the body^7^. And multiple epidemiological studies have confirmed that DEHP exposure is one of the factors that induce asthma, AR, allergic conjunctivitis and other allergic diseases^8–10^. And a study found that DEHP can worsen the nasal environment of AR animal models^11^. Thus, it is closely related to allergic diseases, we hypothesize that DEHP also plays a role in the AR immune regulatory network.

Aryl hydrocarbon receptor (AhR) is a transcription factor that relies on ligand activation, regulating the differentiation, maturation and function of Dendritic Cells (DCs) and exerting powerful immune regulation, which can bind to certain metabolic products in the environment and in the body^12^. Since the immunological characteristics of AR are based on the imbalance of CD4+ Th cell network, which is dominated by the differentiation of Th2 cells, and a study have found that the binding of AhR and its ligand Methyl 2-(1-indole-3-carbonyl)-thiazole-4-carboxylate (ITE) has an important negative regulatory effect on DC cells, which in turn regulates the differentiation and function of downstream Th cells^13^. And Kuo^14^ deems that DEHP can inhibit the secretion of Th1 type cytokines such as IFN, interleukin 12 (IL-12) by PCRs through AhR, thereby inhibiting Th1 type immune response, leading to Th1/Th2 imbalance and promoting the occurrence of allergic diseases. And the main genes that AhR targets are the cytochrome P450 enzymes (CYP), such as CYP1A1 and CYP1B1^15^. Therefore, we speculated that DEHP may be related to AhR-related signaling pathways CYP1A1 and CYP1B1.

DEHP is currently known EDCs, which can have toxic effects on reproductive development, kidneys, lungs, immune system, and nervous system through drinking, eating, skin contact and breathing etc. In recent years, the impact of phthalates on the respiratory tract has become a hot spot. Several studies have suggested that children with asthma and allergic symptoms are related to DEHP^8,10^. Because AR is called “one airway, one disease” together with asthma, its role in AR during DEHP exposure remains to be further explored.

Therefore, to understand the effect of DHEP on AR, our experiment examined DEHP metabolites in urine on AR patients and DEHP gavage method to explore the expression of inflammatory factors, oxidative stress and AhR expression in DEHP exposure alone, DEHP and OVA combined exposure, which base on AR model.

## Materials and methods

### DEHP metabolites detection

According to the ARIA guidelines^1^, we selected 32 patients with AR and 32 subjects without AR (NAR) in the First Affiliated Hospital of Chongqing Medical University. First, we collected basic information of people such as subject name, gender, age, etc., and recorded detailed results of SPT (the skin prick test), and then, collected 10 ml of random middle-stage urine from the subjects, store in clean glass tubes, and freeze at − 20 °C. And we analyzed the correlation between SPT and DEHP metabolite concentration. Then, we used high performance liquid chromatography-mass spectrometry (HPLC–MS) to detect four DEHP metabolites, including MEHHP, MECPP, MEOHP, and MEHP.

### Animal experiments

24 BALB/c mice (male, 6–8 weeks old and 25–30 g) that were specific pathogen-free (SPF) were purchased from the Experimental Animal Center of Chongqing Medical University (Chongqing, China). The mice were housed in individual ventilated cages (IVC) at temperatures between 20 and 25 °C and 50–70% humidity with a 12-h light–dark cycle, which managed by the Experimental Animal Center. The mice were randomly divided into four equal groups with six animals each: (a) a negative control (NC) group; (b) DEHP group; (c) Ovalbumin (OVA) group; (d) OVA and DEHP group.

**Figure 1:**
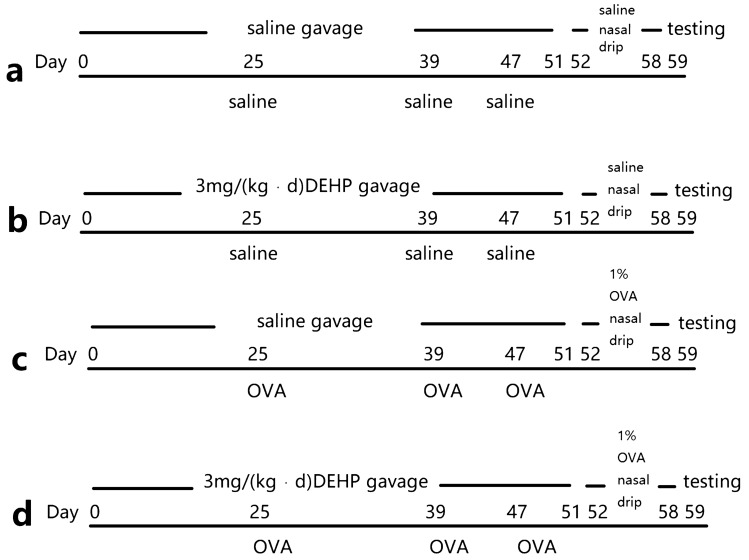
(**a**) NC group; (**b**) DEHP group; (**c**) OVA group; (**d**) OVA and DEHP group.

We keep the BALB/c mice free to drink and eat, and they entered the experiment after 1 week of adaptive feeding, starting DEHP (Sigma, USA) gavaged as the first day. The mice were gavaged with saline or DEHP (3 mg/kg/body) from day 1 to 51 (51 times), and were sensitized with OVA and Al(OH)_3_ (0.5 mg/ml OVA (Sigma, USA) in saline and 20 mg/ml (2%) Al(OH)_3_ gel each time) or saline (isometric saline each time) by intraperitoneal injection on day 25, 39 and 47. This was then followed by a nasal drip in 1% OVA (20 μl per side nasal each time) from days 52 to 58 (7 times) using a pipette (Eppendorf, Germany)^16^. The detailed protocol outline is shown in Fig. 1.

In order to ensure the integrity of the nasal mucosa, we immediately cut the skin of the mouse's head after eyeball blood collection, removing the entire nasal cavity and then decalcify. After decalcification, it is used for Hematoxylin and Eosin (H&E) staining after fixation with 4% paraformaldehyde (Sangon Biotech, Shanghai, China). Another nasal mucosa tissue was washed in Phosphate Buffer Saline (PBS) (4 °C) and stored at − 80 °C.

### Allergic symptom scores

After the nasal drip, the mice will have symptoms such as sneezing, scratching nose and runny nose. We have statistics on it. And standard is as follows, observe for 15 min after the last nasal drop, 1 to 2 sneezes are counted as 1 point, 3 to 5 are counted as 2 points, and 6 or more are counted as 3 points; the light rubbing nose is counted as 1 point, the scratching of the nose and the face is 2 points, and 3 points for scratching everywhere; scratching 1 to 2 times is counted as 1 point, scratching 3 to 5 times is counted as 2 points, 6 times or more counted as 3 points; the runny nose to the front nostril is counted as 1 point, beyond the front nostril is 2 points, and the nose flow in face is 3 points.. The superposition method records the total score. And if the total score exceeds 7 points, the AR mouse model is successfully constructed^17^.

### Nasal mucosal section, tissue fixation, and staining

After the mice were euthanized in each group on NC, DEHP, OVA and OVA + DEHP, the entire nasal cavity of mice were cut. Then the nasal cavities were immersed with 4% neutral buffered formaldehyde and fixed overnight at room temperature and then decalcified. Tissues were embedded in paraffin, sectioned, and stained with HE-staining.

### Enzyme-linked immunosorbent assay (ELISA)

After thawed the preserved mouse serum to room temperature, IFN-γ, Mouse total serum IgE, IL-4, IL-5, IL-6, IL-13 and IL-33 levels in the serum were measured using IFN-γ ELISA Kit (4A Biotech, Beijing, China), Mouse total serum IgE ELISA Kit (4A Biotech), IL-4 ELISA Kit (4A Biotech), IL-5 ELISA Kit (4A Biotech), IL-6 ELISA Kit (4A Biotech), IL-13 ELISA Kit (4A Biotech) and IL-33 ELISA Kit (4A Biotech), respectively, in accordance with manufacturers’ instructions.

### Reactive oxygen species assay

DCFH-DA (2,7-dichlorofuorescin diacetate) is the most commonly used and sensitive intracellular reactive oxygen species(ROS) detection probe. A ROS Assay Kit (Beyotime, Hainan, China) was used to survey intracellular levels of ROS in the nasal mucosa cells. And DCFH-DA was added to the cell culture at a final concentration of 10 μM for 45 min (37 °C). Then, cells labeled with incubation probe were collected and washed with PBS. Total fluorescence intensity was detected with a Microplate Reader + thermo + (Varioskan + LUX, ThermoFisher, USA) with excitation and emission wavelengths of 500 nm and 525 nm, respectively. Protein concentration was determined with Bicinchoninic Acid protein assay kit (Beyotime) and with wavelengths of 562 nm.

### Oxidative stress and antioxidant enzyme assays

The superoxide dismutase (SOD), Malondialdehyde (MDA) and glutathione peroxidase (GSH-Px) activities were measured with total-SOD (T-SOD), MDA and GSH-Px assay kits (Jiancheng Bioengineering, Nanjing, China), according to manufacturer’s instructions. Briefly, the nasal mucosa cells were added 9 volumes of saline according to the weight of tissue (g): volume (ml) = 1:9, and mechanically homogenize in ice water bath to prepare 10% homogenate, then supernatant detected after centrifugation. Protein concentration was determined with Bicinchoninic Acid protein assay kit, and enzyme activity was standardized, a standard curve to drew for albumin from bovine serum (BSA) standard solutions.

### Western blotting analysis

First, take blood from the mouse eyeball and then obtain monocytes. A nuclear and cytoplasmic protein extraction kit (Beyotime) was used to purify the nuclear proteins and cytoplasmic proteins of peripheral blood cells of mice. AhR and GAPDH were evaluated by western blot analysis as described. Anti-AhR primary antibodies were purchased from Abcam (USA) and anti-GAPDH antibodies were purchased from Abcam. Relative expression levels were quantified using Quantity One software, version 4.52 (Bio-Rad)^18^.

### Real-time quantitative reverse transcription PCR (qRT-PCR)

After animal experiments, we cultured peripheral blood cells of mice. The AhR, CYP1A1 and CYP1B1 mRNA expression were detected by real-time qRT-PCR as previously described. Total RNA of peripheral blood cells of mice was extracted using TRIzol reagent (Beyotime, China), and reverse transcribed into cDNA. The synthesized cDNA was then quantifed by SYBR Green (Western Biotechnology, Chongqing, China) assay on a Real-Time Detection System (Funglyn Biotech, Canada). Actin was used as an endogenous reference for mRNAs. All primers were obtained from Western Biotechnology, and in Table 1, their sequences are presented. And verify mRNA by calculating relative expression and relative ratio^13^.

**Table 1:** Primer sequences used for qRT-PCR.

Symbol (base pair)	Primer (5ʹ to 3ʹ)
AhR (149 bp)	Forward: CCTACCAATACGCACCAAAAG Reverse: CAGGGCTTGAAGGAGGACAC
CYP1A1 (135 bp)	Forward: GACCCTTACAAGTATTTGGTCGTG Reverse: GCCAGTAACCTCCCCAAACTC
CYP1B1 (159 bp)	Forward: GCTTCGGCTGTCGGTACAAC Reverse: CGAACTTGCGGAAGGTGG
Actin (263 bp)	Forward: GAGACCTTCAACACCCCAGC Reverse: ATGTCACGCACGATTTCCC

### Statistical analysis

Statistical analysis was performed using SPSS software for Windows (version 22.0; SPSS, Inc., Chicago, IL, USA) and GraphPad Software (version 7.0; San Diego, CA, USA). Results were analyzed using analysis of variance (ANOVA) (Tukey test), and data were shown as the mean ± standard deviation (SD) if it conformed to a normal distribution. Otherwise, results were analyzed using Mann–Whitney U test, and data were shown as the median and geometric mean (GM). The P-values less than 0.05 were considered significant.

### Ethics statement

All experiments involving animals and tissue samples were performed in accordance with the guidelines of the National Institutes of Health (NIH) and Chongqing Medical University with all procedures approved by the Institutional Animal Care and Use Committee of Chongqing Medical University (Chongqing, China).

The Ethics Committee of Chongqing Medical University approved this study and all subjects provided their written informed consent to participate in the study. 32 AR patients (18 female, 14 male) and 32 healthy controls (17 female, 15 male) were included in the study.

## Results

### Detection of DEHP metabolites

We enrolled 64 subjects included males and females, aged between 9 and 66 years. We recorded the positive results of SPT house dust mite and dust mite in patients with AR and analyzed their correlation with four metabolites of DEHP by Spearman. All the results were p > 0.05, and there was no significant correlation between the concentration of metabolites and the severity of allergy. The reason might be that the metabolites are affected by many factors. Except that the detection rate of MECPP was 61.1% and MEHP was 83.3%, the detection rates of MEHHP and MEOHP were both 100% in Table 2. The concentration of MEHHP, MECPP, MEOHP and MEHP in the urine of the subjects was skewed in this study, and the differences of MEHHP, MEOHP and MEHP concentrations between AR group and NAR group were statistically significant (P < 0.05), and the MECPP concentrations were not significantly different between the two groups (P > 0.05).

**Table 2:** Distribution of DEHP metabolites of AR group and NAR group (ng/ml).

DEHP metabolites	The detection rate (%)	AR		NAR	
		Median (P25,P75)	Geometric mean	Median (P25,P75)	Geometric mean
MEHHP	100	5.60 (2.06,12.92)	8.73	3.10 (1.38,4.93)	3.13
MECPP	61.1	5.99 (1.73,10.83)	8.02	4.79 (1.19,8.39)	4.79
MEOHP	100	4.70 (1.71,11.09)	6.82	2.40 (1.50,3.61)	2.51
MEHP	83.3	1.65 (0.86,3.64)	2.71	0.35 (0.02,2.06)	0.81

### Allergic symptom scores of AR model

Allergic symptom scores were consisted of the numbers of sneezing, scratch, the numbers of scratching and runny nose per 15 min. And OVA and OVA + DEHP group exhibited significantly higher allergic symptom scores than NC and DEHP group (Table 3).The data of scores are shown as the mean ± SD, p < 0.05.

**Table 3:** Allergic symptom scores, data are mean ± SD, n = 6 per group.

Group	Sneeze	Scratch	The number of scratching	The nose flow	Total scores
NC	0.50 ± 0.22	0.33 ± 0.21	0.33 ± 0.21	0.17 ± 0.17	1.33 ± 0.56
DEHP	0.50 ± 0.22	0.50 ± 0.22	0.67 ± 0.33	0.33 ± 0.21	2 ± 0.93
OVA	2.17 ± 0.31	2.33 ± 0.21	2.33 ± 0.21	2.17 ± 0.17	9 ± 0.82
OVA + DEHP	2.50 ± 0.22	2.83 ± 0.17	2.67 ± 0.21	2.50 ± 0.22	10.50 ± 0.50

### Analysis of nasal mucosa histology and mouse total serum IgE

**Figure 2:**
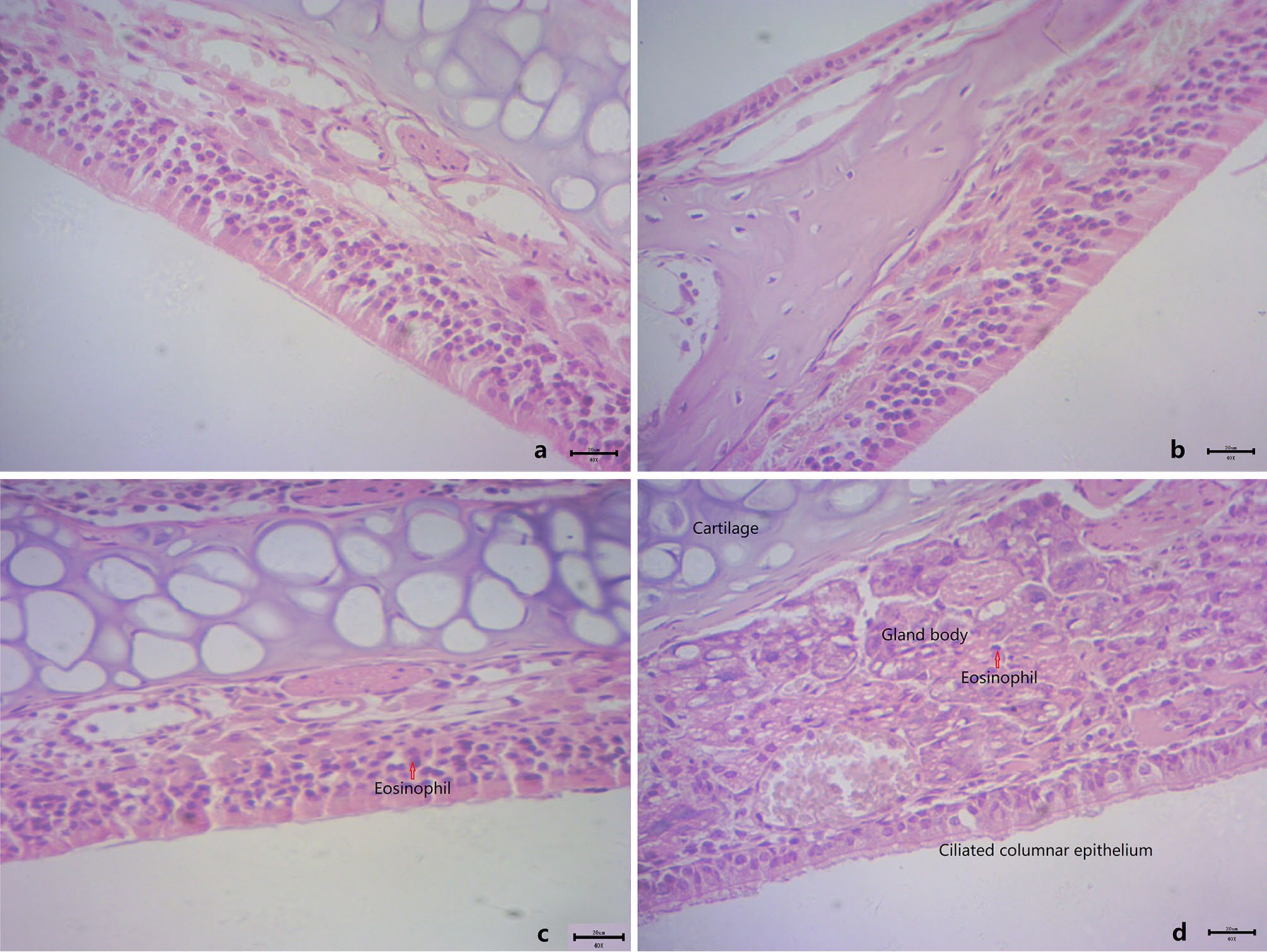
The effects of OVA/DEHP exposure on nasal mucosa histology damage demonstrated with HE-staining. (**a**) NC group; (**b**) DEHP group; (**c**) OVA group; and (**d**) OVA + DEHP group (original magnification: (**a**–**d**), × 400, Scale bars = 20 μm).

The nasal mucosa from the NC (Fig. 2a) and DEHP (Fig. 2b) group showed no histopathological abnormalities in HE-staining. However, the nasal mucosa of exposure to OVA (Fig. 2c) and OVA + DEHP (Fig. 2d) group showed different degrees morphological alterations. The nasal mucosa from the NC group and DEHP group showed normal morphology. And then eosinophil infiltration could be seen and the tissue structure was loose, the arrangement of ciliated columnar epithelial cells were disordered of nasal mucosal in the OVA group (Fig. 2c) and OVA + DEHP group (Fig. 2d).

**Figure 3:**
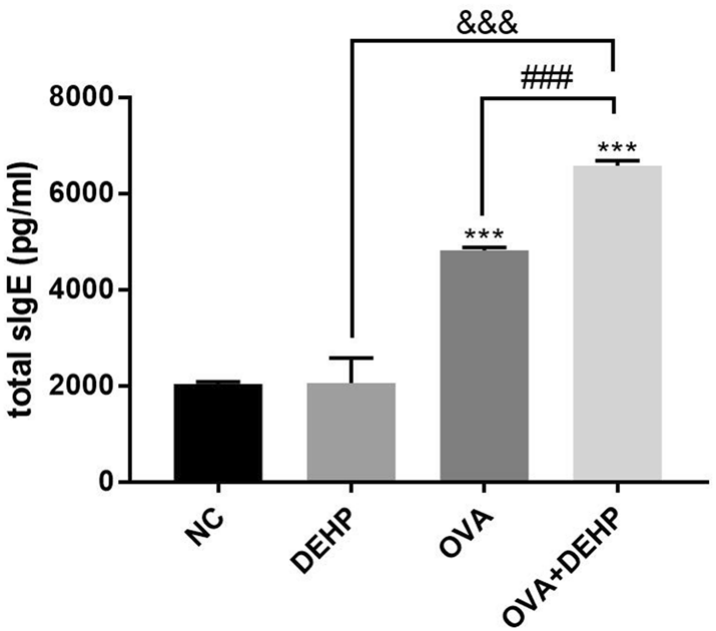
Total serum IgE concentrations (pg/ml) in the 4 different exposure groups. Data are mean ± SD, (n = 6), *p < 0.05, **p < 0.01, ***p < 0.005, compared with the saline control; ^&^p < 0.05, ^&&^p < 0.01, ^&&&^p < 0.005, compared with the DEHP only group; ^#^p < 0.05, ^##^p < 0.01, ^###^p < 0.005, compared with the OVA only group.

The Fig. 3 described the total serum IgE for all experimental groups. It was not difficult to find that the total serum IgE increased significantly in the OVA group and the OVA + DEHP group, and its level of OVA + DEHP group were significantly raised in relation to OVA only exposure group. The total serum IgE of the DEHP group was not significant difference compared with the NC group. All p less than 0.01 by statistical analysis.

### Effects of DEHP on the levels of inflammatory cytokines in the serum of mice

**Figure 4:**
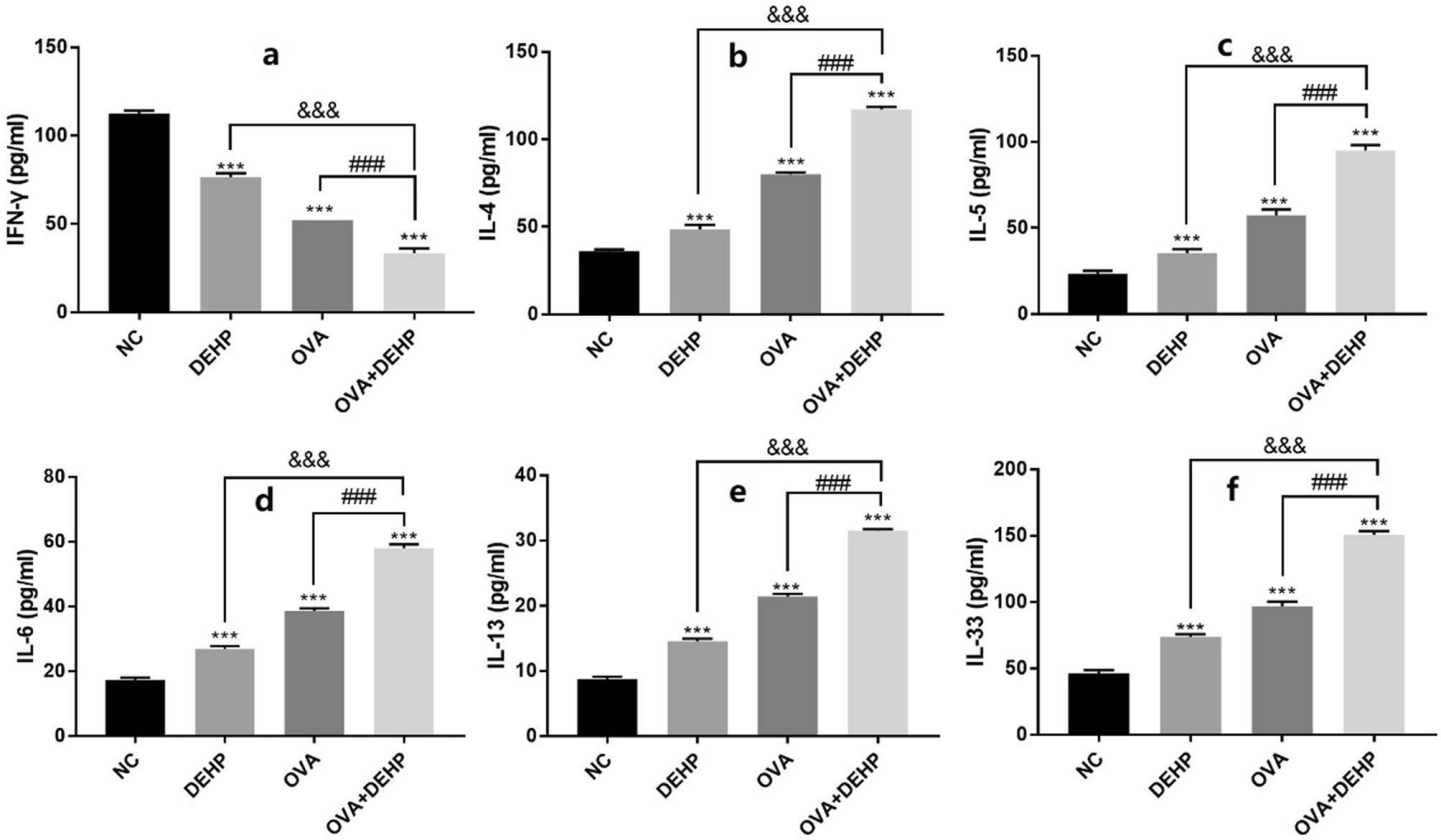
DEHP stimulates the production of inflammatory cytokines in BALB/c mice`s serum. The levels of IFN-γ (**a**), IL-4 (**b**), IL-5 (**c**), IL-6 (**d**), IL-13 (**e**) and IL-33 (**f**) were determined with ELISA. All data are presented as the mean ± SD, (n = 6), *p < 0.05, **p < 0.01, ***p < 0.005, compared with the saline control; ^&^p < 0.05, ^&&^p < 0.01, ^&&&^p < 0.005, compared with the DEHP only group; ^#^p < 0.05, ^##^p < 0.01, ^###^p < 0.005, compared with the OVA only group.

In order to examine the impact of DEHP on inflammatory cytokines production, the levels of IFN-γ, IL-4, IL-5, IL-6, IL-13 and IL-33 were measured by ELISA. And IL-4, IL-5, IL-6, IL-13 and IL-33 levels significantly increased in BALB/c mice`s serum treated in experimental groups when compared with NC group, in addition, IFN-γ declined in expression. And the levels of all the above inflammatory factors in OVA + DEHP group were significantly raised in relation to OVA only exposure group except IFN-γ. (Fig. 4).

### Effects of DEHP on ROS, GSH-Px, SOD and MDA levels in the nasal mucosa of mice

**Figure 5:**
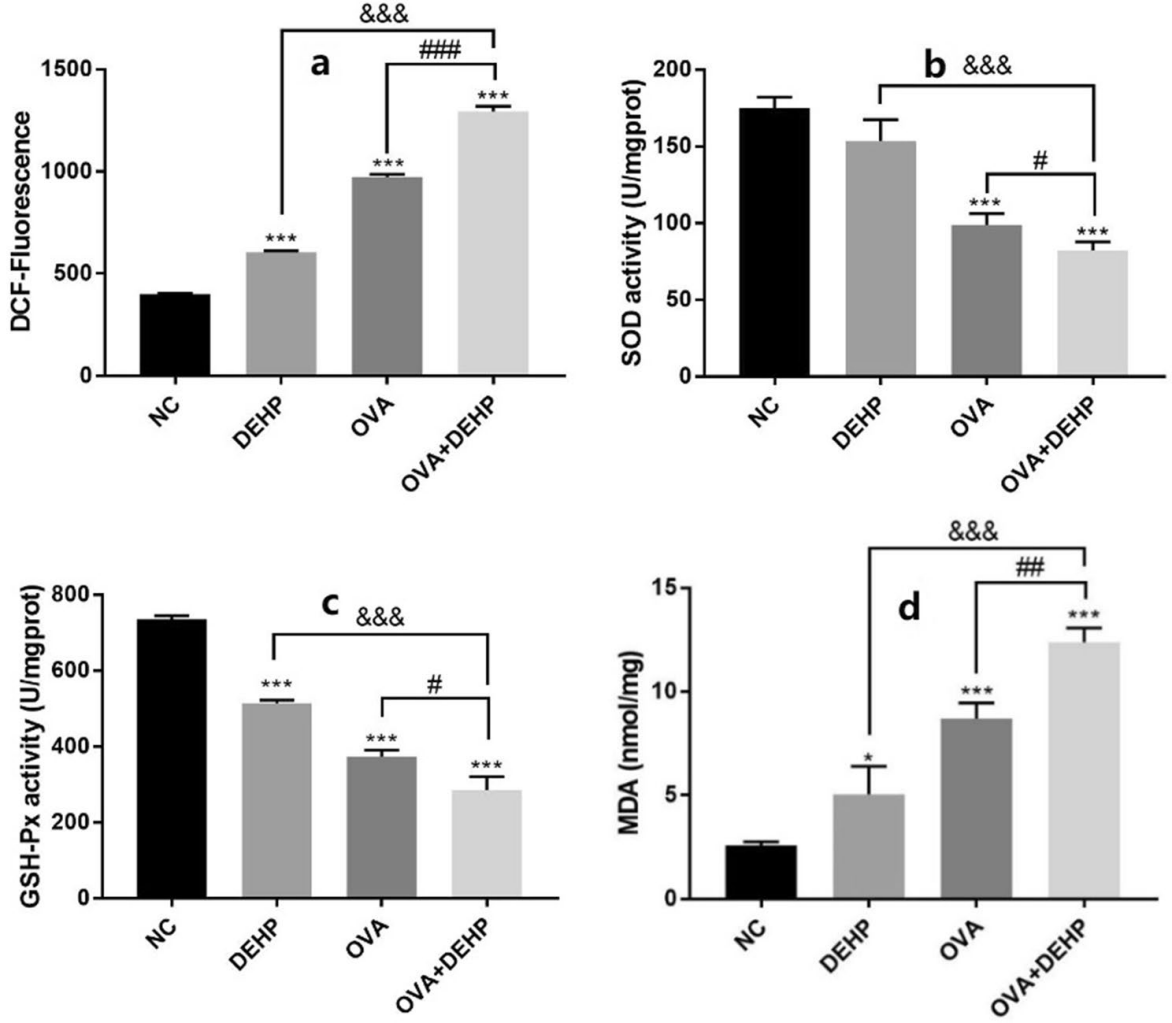
DEHP induces oxidative stress in nasal mucosa cells. (**a**) Levels of ROS induced by DEHP. (**b**,**c**) Glutathione peroxidase (GSH-Px) and activities of superoxide dismutase (SOD) in nasal mucosa cells were affected by DEHP, (**d**) content of Malondialdehyde (MDA) in nasal mucosa cells were increased. (n = 6), Data are mean ± SD, *p < 0.05, **p < 0.01, ***p < 0.005, compared with the saline control; ^&^p < 0.05, ^&&^p < 0.01, ^&&&^p < 0.005, compared with the DEHP only group; ^#^p < 0.05, ^##^p < 0.01, ^###^p < 0.005, compared with the OVA only group.

In order to examine the impact of DEHP on oxidative stress in the nasal mucosa cells. We used nasal mucosa cells to detect oxidative stress, including ROS, SOD, GSH-Px and MDA, and levels of ROS were determined by DCFH-DA (2′,7′-Dichlorofluorescin diacetate) fluorescence intensity assay. The DEHP significantly elevated ROS levels when cooperated with OVA to induce oxidative stress (Fig. 5a). And the activities of SOD and GSH-Px decreased, but we did not find a correlation between DEHP and SOD activity (Fig. 5b,c). Moreover, compared with the NC group, the concentrations of MDA in the DEHP group, the OVA group, and the OVA + DEHP group increased, and the OVA + DEHP group was more significant (Fig. 5d).

### Effects of DEHP on mice AhR protein and relational expression

**Figure 6:**
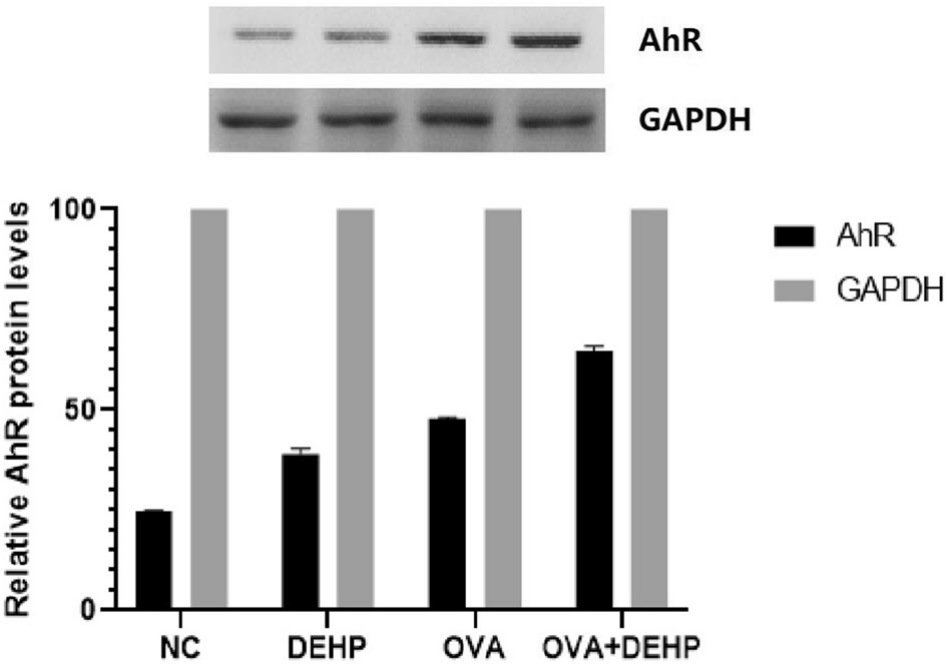
Quantitative analysis of the effects of OVA/DEHP on the expression of AhR. The values are presented as the means ± SD, (n = 6), p < 0.05.

**Figure 7:**
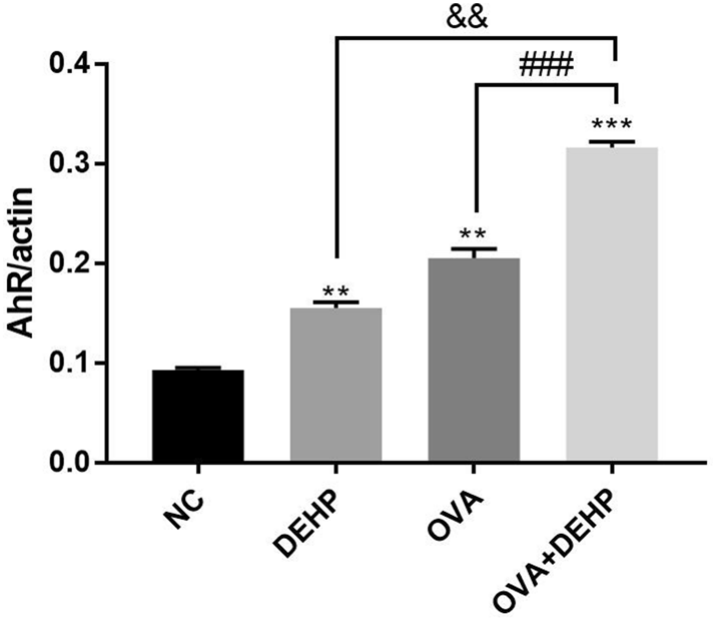
The mRNA expression levels of AhR was measured by qRT-PCR analysis. The expression levels of mRNA were normalized to actin and given as fold induction compared to the mRNA level in control cells. All data are presented as the mean ± SD, (n = 6), *p < 0.05, **p < 0.01, ***p < 0.005, compared with the saline control; ^&^p < 0.05, ^&&^p < 0.01, ^&&&^p < 0.005, compared with the DEHP only group; ^#^p < 0.05, ^##^p < 0.01, ^###^p < 0.005, compared with the OVA only group.

The protein (Fig. 6) and mRNA levels (Fig. 7) of AhR were significantly increased in DEHP, OVA and OVA + DEHP exposure groups when compared with NC group, and with respect to the OVA alone group and the DEHP alone group , OVA + DEHP group is more significant.

**Figure 8:**
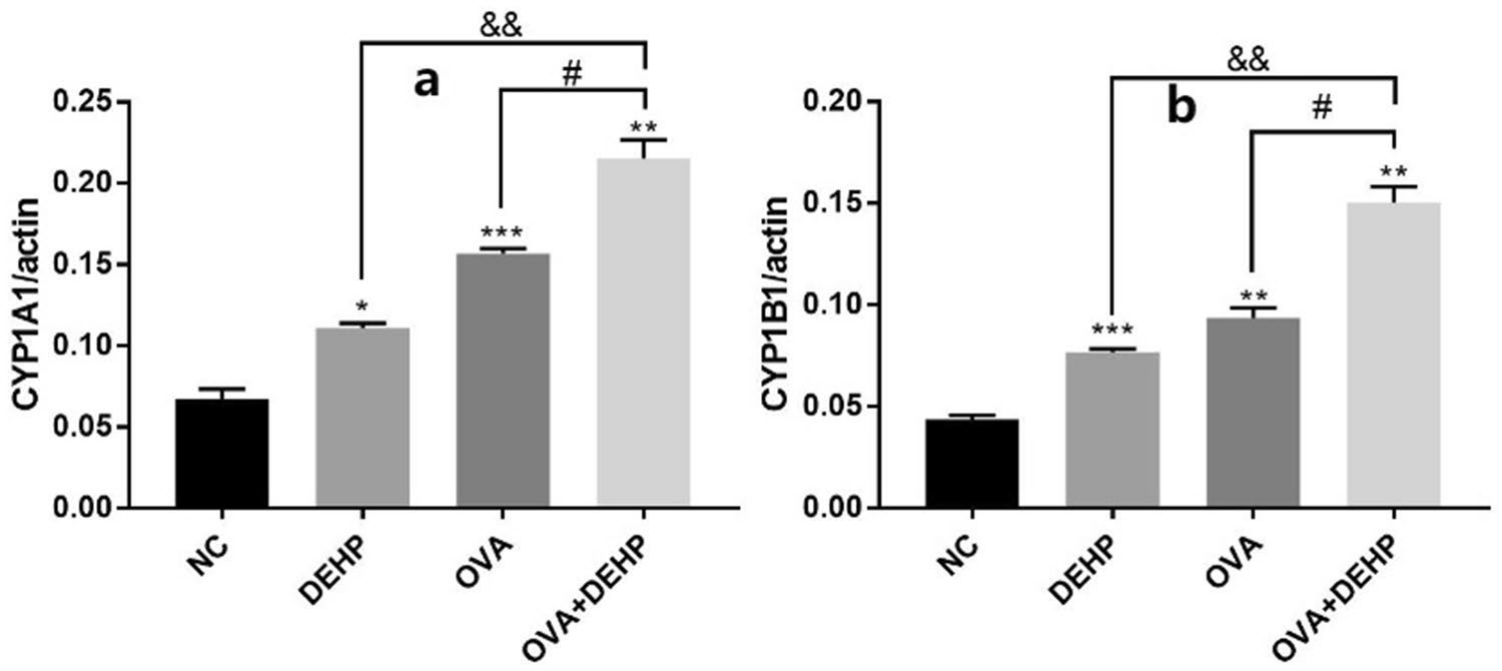
The mRNA expression levels of CYP1A1 (**a**) and CYP1B1 (**b**) were measured by qRT-PCR analysis. The expression levels of mRNA were normalized to actin and given as fold induction compared to the mRNA level in control cells. All data are presented as the mean ± SD, (n = 6), *p < 0.05, **p < 0.01, ***p < 0.005, compared with the saline control; ^&^p < 0.05, ^&&^p < 0.01, ^&&&^p < 0.005, compared with the DEHP only group; ^#^p < 0.05, ^##^p < 0.01, ^###^p < 0.005, compared with the OVA only group.

The mRNA expression levels of CYP1A1 and CYP1B1 were measured by qRT-PCR analysis. The CYP1A1 and CYP1B1 both are associated with the signaling pathway of AhR, and we found that their levels in the only DEHP, only OVA and OVA + DEHP exposure groups had significant differences compared with the NC group. Moreover, the OVA + DEHP group increased significantly compared with the others (Fig. 8).

## Discussion

In our study, we found that the concentrations of DEHP metabolites MEHHP, MEOHP, and MEHP in the urine of patients with AR were significantly higher than those in the NAR group, but MECPP was not. More than 3 kinds of DEHP metabolites could be found more in AR patients, therefore, we believed that the DEHP exposure in patients with AR is higher than that in the NAR group, so we assumed that DEHP might have some effect on the mechanism of AR and designed the animal experiment.

A research^19^ demonstrated that DEHP intensifies atopic dermatitis in NC/Nga mice, which identified a positive biological link between DEHP exposure and the facilitation of allergic pathophysiology. Larsen^20^ also showed that DEHP lead allergic asthma to worse in BALB/c mice. However, did this EDCs affect AR, which was also an allergic disease? In the present study, we refered to the study^11^ on asthma, which exposure to DEHP (3 mg/kg/body) did significantly deteriorate related nasal inflammatory response in AR BALB/c mice, and histological findings were slightly worsened in the OVA + DEHP group than in the only OVA group. These findings were somewhat different on AR that might be due to the experimental agreement used, such as the DEHP dosage, exposure design and/or sample material. Therefore, our study might have different discoveries in AR model of exposing DEHP.

First, we established the animal model, and found that OVA and OVA + DEHP group both were successful AR model by means of allergic symptom scores, histological changes and total serum IgE. And then, the mice’s serum detected, including the levels of IFN-γ, IL-4, IL-5, IL-6, IL-13 and IL-33. We discovered that the levels of IFN-γ inhibited by DEHP, which it may be relate to Th1/Th2 equilibrium. And diisononyl phthalate (DINP is a member of the phthalate family) was shown to suppress the polarization of Th1 and enhance the polarization of Th2 in Hwang’s study^21^. You^22^ detected some cytokines in bronchoalveolar lavage fluid (BALF) in asthma model, and OVA and DEHP participated a salient Th2 response which was characterized by the upregulation of Th2-type cytokines, such as IL-4, IL-5 and IL-13. However, they considered that OVA had a greater impact than DEHP in this study, which was a little inconsistent with us who considered that DEHP may act as an adjuvant. The reason may be due to different animal models and samples. Then we discovered that DEHP increased the expression of IL-6 and IL-33, similarly, Wang^23^ revealed that DEHP can via NF-kB mediated up regulation of IL-6, and Ashley-Martin^24^ also found that prenatal exposure to phthalates increased level of IL-33.

According to cytokine results by ELISA, and that could find this outcome which OVA + DEHP group was more influential than only OVA group and DEHP group, it was consistent with allergy symptom scores, histological changes, and total serum IgE results. And we also tested oxidative stress with nasal mucosa cells. Similarly, we found that ROS and MDA levels were higher in the OVA + DEHP group than in the OVA group and DEHP group, and activities of GSH-Px and SOD suppressed, OVA + DEHP group is also lower. And this result was similar to that of You’s^22^. So, we considered that DEHP can aggravate the nasal inflammatory response caused by OVA and promote the Th2 response of AR.

AhR is a transcription factor exerting powerful immune regulation which can bind to certain metabolic products in the environment and in the body. And phthalates have been accepted as exhibiting a weak potency as agonists of AhR^25^. Recent a research^26^ found that DEHP involved an impairment in AhR signaling in mouse cerebral cells. These data support our hypothesis that DEHP-promoted was related with AhR. Our data showed that AhR mRNA expression did change in answer to DEHP-promoted, simultaneously, AhR protein expression increased too. A similar tendency was observed regarding CYP1A1 and CYP1B1 mRNA expression. These data had shown that DEHP- promoted could enhance AhR protein and AhR, CYP1A1 and CYP1B1 mRNA expression, which the adjuvant effect of DEHP might be achieved through this approach. And then, Du^27^ reported identical results when they observed DEHP-induced cerebellar toxicity in Coturnix japonica by disrupting the CYP enzyme system homeostasis. The author demonstrated an increase in both AhR and CYP1B1 mRNA expression with DEHP. Therefore, in our opinion, DEHP shows an adjuvant effect, possibly, through the AhR signaling pathways CYP1A1 and CYP1B1, which are closely related to the environment (Supplementary Information 1).

## Conclusion

By detecting DEHP in urine, we found that the concentrations of 3 metabolites of DEHP (MEHHP, MEOHP, and MEHP) in urine of AR patients were higher, DEHP that entering the body might have a certain effect on the immune system. In AR model, our results illustrated that DEHP helped AR mice to increase Th2 response, oxidative stress and cause mucosal damage in nasal mucosal cells. And that these were attributed to the adjuvant effect of DEHP which might pass through the CYP1A1 and CYP1B1 signaling pathways of AhR.

## Supplementary information

Supplementary Information 1.
