# Supplementary material for: Effect of di-(2-ethylhexyl) phthalate (DEHP) on allergic rhinitis
Source: Sci Rep. 2020 Sep 3;10:14625. doi: 10.1038/s41598-020-71517-6 (PMC7471965; doi:10.1038/s41598-020-71517-6)
Supplement: Supplementary file 1 — Supplementary Information 1. [file 41598_2020_71517_MOESM1_ESM.zip › WB-AhR.pdf]

Effect of di-(2-ethylhexyl) phthalate (DEHP) on Allergic Rhinitis

Qi-Yuan Zou[1*], Su-Ling Hong[1*], Hou-Yong Kang[1], Xia Ke[1], Xiao-Qiang Wang[1], Jia Li[1], Yang Shen[1]

* Contributed equally

1.Department of Otorhinolaryngology, The First Affiliated Hospital of Chongqing Medical University, Chongqing, People's Republic of China

E-mail:

Qi-Yuan Zou: zouqiyuan1992@sina.com

Su-Ling Hong: hsl_prof@163.com

Hou-Yong Kang: kanghouyong@sohu.com

Xia Ke: drkexia@163.com

Xiao-Qiang Wang: 153895639@qq.com

Jia Li: 247738117@qq.com

Correspondence to Dr. Yang Shen, E-mail: sy_smile@sina.cn

Phone: 86-015111953398　　Fax: 86-02389012695

Address: Department of Otorhinolaryngology, The First Affiliated Hospital of Chongqing Medical University, 1#Yixueyuan Road, Chongqing 400016, People's Republic of China

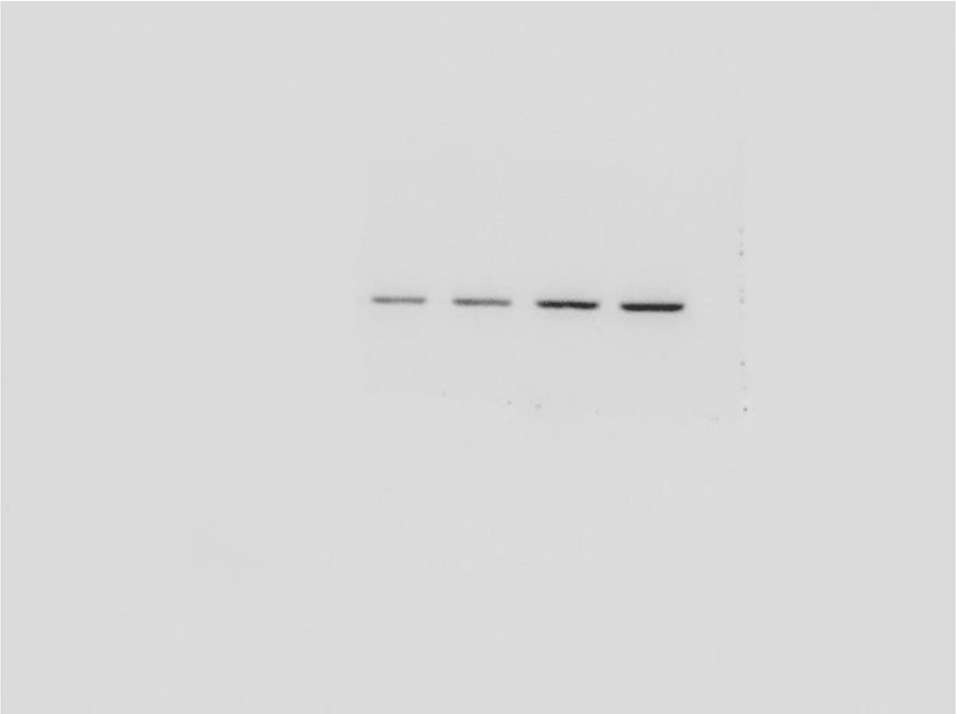

WB-AhR
